# Doping effect and oxygen defects boost room temperature ferromagnetism of Co-doped ZnO nanoparticles: experimental and theoretical studies†

**DOI:** 10.1039/c9ra03620b

**Published:** 2019-07-25

**Authors:** Yan Zong, Yong Sun, Shiyan Meng, Yajing Wang, Hongna Xing, Xinghua Li, Xinliang Zheng

**Affiliations:** School of Physics, Northwest University, Xi'an 710069 China xinghua.li@nwu.edu.cn

## Abstract

Co-doped ZnO nanoparticles with different dosage concentrations were fabricated by a thermal decomposition method. The nanoparticles show a pure wurtzite structure without the formation of a secondary phase or Co clusters, in which Co ions present as Co^2+^ and occupy Zn^2+^ tetrahedral sites within the ZnO matrix. All the samples show ferromagnetic properties at room temperature with nonzero coercivity and remanence magnetization. Besides, the magnetic data is also fitted by the model of bound magnetic polarons (BMP). By increasing the Co^2+^ doping concentration, the saturation magnetization values of Co-doped ZnO nanoparticles increase first and then decreases, which is related to the variation tendency of oxygen defects 
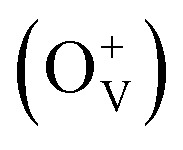
 on the surface and the number of BMPs. This phenomenon can be ascribed to the formation of defect-induced BMPs, in which 
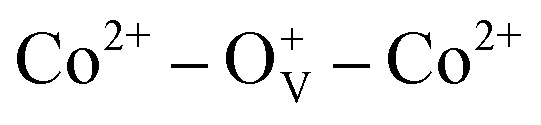
 ferromagnetic coupling occurs at lower Co^2+^ concentration and Co^2+^–O^2−^–Co^2+^ antiferromagnetic coupling arises at higher Co^2+^ concentration. Air annealing experiments further demonstrate this result, in which the saturation magnetization of Co-doped ZnO nanoparticles is reduced after annealing in Air. The doping effect and oxygen defects on the magnetic ordering of Co-doped ZnO were calculated using density functional theory. The calculation results reveal that stable long-range magnetic ordering in Co-doped ZnO nanoparticles is mainly attributed to the localized spin moments from 3d electrons of Co^2+^ ions. Both the experimental and theoretical studies demonstrate that the ferromagnetism in Co-doped ZnO nanoparticles is originated from the combined effects of Co doping and oxygen vacancies. These results provide an experimental and theoretical view to understand the magnetic origination and tune the magnetic properties of diluted magnetic semiconductors, which is of great significance for spintronics.

## Abstract 2

Co-doped ZnO nanoparticles with different dosage concentrations were fabricated by a thermal decomposition method.

## Graphical abstract

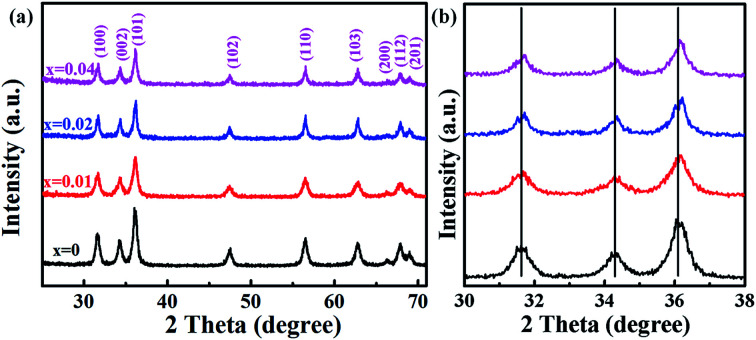

## Introduction

ZnO, as a wide-band semiconductor, has been a fascinating host of dilute magnetic semiconductors (DMSs) due to its electronic-controlled spin properties and potential applications in spin-electronic devices.^1–3^ For practical applications, the demanded high data processing speeds and large integration densities in spin-electronic devices urgently require room-temperature ferromagnetic behavior with large magnetism.^4^ However, there are some overwhelming roadblocks related to its n-type conductivity and origin of intrinsic ferromagnetism for DMSs, which restrict its long-term development and applications. One feasible project is to regulate and control defects on the surface of materials,^5–11^ the other is doping 3d magnetic transition-metal ions (such as Fe, Co, Ni, Mn *etc.*) into the ZnO lattice.^12–18^ These magnetic transition-metal ions have partially filled d orbit with unpaired spin electrons, which can not only provide net magnetic moment, but also tailor the position of Fermi energy level and further tune the optical/electric properties of ZnO host. Incorporating 3d magnetic transition metal ions into ZnO lattice has been a common strategy for constructing novel DMSs with high ferromagnetism at room temperature.

Among the 3d magnetic transition metal ions, Co^2+^ is promised to be a fascinating doping element to explore novel ZnO-based DMSs with high room temperature ferromagnetism. First, the ionic radius of Co^2+^ (0.72 Å) is similar to that of Zn^2+^ (0.74 Å). Co^2+^ can easily substitute the Zn^2+^ ions within ZnO lattice and form solid solution, which can avoid the lattice mismatch and maintain the crystalline structure of ZnO.^15,17,19^ Second, Co has a large atomic magnetic moment (*μ*_Co_ = 1.8 *μ*_B_) with high Curie temperature of 1388 K.^20^ Third, Co has rich electronic states which can adjust the magnetic and optical properties of ZnO.^19,21,22^ During the past decades, Co-doped ZnO nanostructures with various morphologies have been prepared by different methods.^23,24^ However, it should be notice that the magnetic properties of Co-doped ZnO reported by different groups are quite incongruous. The intrinsic nature of magnetic ordering in Co-doped ZnO is still not clear, which is probably affected by the different morphologies and fabricated methods.^19,25–28^ For instance, Ji Hongfen *et al.* reported that the room-temperature ferromagnetism of Co-doped ZnO nanoparticles is ascribed to the joint effects of bound magnetic polarons and intrinsic exchange-interactions;^19^ Verma K. C. reported that ZnO nanoparticles doped by low concentrated Co ions were fabricated *via* a sol–gel method, and long-range ferromagnetic ordering were induced by free charge carriers and lattice oxygen vacancies.^26^ Moreover, lots of other models, such as carrier-mediated exchange,^29^ double exchange,^30^ super exchange,^31^ F-center exchange,^32^ bound magnetic polarons^33,34^ and Zener/RKKY,^35^ were used to explain the original mechanism of ferromagnetism. However, the Co-doped ZnO nanostructures reported by the above method are usually aggregated or the Co dopants may greatly affect the size and shape of ZnO host, which make the ferromagnetism investigations more complex. Therefore, the fabrication of Co-doped ZnO nanoparticles with little size difference and deeply understand the intrinsic nature of ferromagnetism are imminently needed.

In this paper, Co-doped ZnO nanoparticles with different doping Co concentrations were synthesized by thermal decomposition method. Both the experimental and theoretical tools were used to study the magnetic properties. We aim to figure out the intrinsic origination of ferromagnetism in DMSs from an experimental/theoretical view, and open up a construction way for the exploration of spin-electronic devices.

## Experimental section

### Materials

Benzyl ether (C_12_H_10_O, 98%), oleic acid (C_18_H_34_O_2_, 90%) and cobalt acetylacetonate (Co(acac)_2_) were purchased from Alfa Aesar. Oleylamine (C_18_H_37_N, 50%) was acquired from TCI. 1-Octadecene (C_18_H_36_, 90%) and zinc acetylacetonate (Zn(acac)_2_) were bought from Acros Organics. 1,2-Decanediol was obtained from Aldrich. All the chemical regents in the experimental process were used as received without further purification.

### Synthesis of Co-doped ZnO nanoparticles

Undoped and Co-doped ZnO nanoparticles (Zn_1−*x*_Co_*x*_O, 0 ≤ *x* ≤ 0.04) were fabricated *via* a thermal decomposition method. 6 mmol of oleic acid, 6 mmol of oleylamine, 8 mmol of 1,2-dodecanediol, 2 − 2*x* mmol of Zn(acac)_2_ and 2*x* mmol of Co(acac)_2_ (0 ≤ *x* ≤ 0.04) were gradually dissolved in 10 ml 1-octadecylene and 10 ml benzyl ether. Under magnetically stirring, the gray-brown mixture solution was dehydrated at 120 °C for 1 h, during which the color gradually converts into brilliant brown. Second, the solution was heated from 120 to 200 °C within 6 min and maintained for 1 h. Finally, the brilliant brown solution was heated to 300 °C with a heating rate of 10 °C min^−1^ and kept at this temperature for 30 min. During all the process, Ar was used as the protective gas. When the reaction was finished, 20 ml of isopropyl alcohol was added into the above solution. The products were centrifuged at 10 000 rpm for 3 min, and washed by hexane/isopropyl alcohol (1 : 1 vol) for 3–5 times.

### Structure characterization

The structures of samples were performed by X-ray powder diffraction (XRD, DX-2700, Cu Kα radiation). The morphologies were characterized by transmission electron microscope (TEM, FEI Tecnai G^2^ F20). The element states and its related bonding characteristics were measured by X-ray photoelectron spectroscopy (XPS, ESCALAB210). The elemental contents of Co and Zn were measured by inductively coupled plasma atomic emission spectroscopy (ICP-AES, ICAP 6300 Series). The magnetic properties were performed by vibrating sample magnetometer (VSM, Lake Shore 7304).

## Results and discussions

**Fig. 1:**
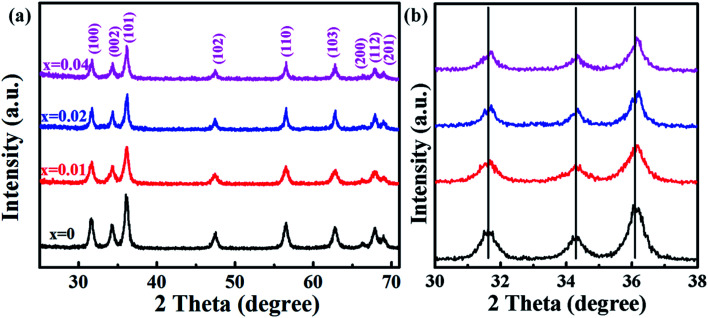
(a) XRD patterns of Zn_1−*x*_Co_*x*_O nanoparticles (0 ≤ *x* ≤ 0.04); (b) magnified part of the three predominant (100), (002) and (101) peaks.

Fig. 1(a) shows the XRD patterns of undoped and Co-doped ZnO nanoparticles with different Co doping concentrations. The positions and relative intensities of all the diffraction peaks can be well indexed into a single-phase hexagonal wurtzite structure of ZnO with space group of *P*6_3_*mc* (186), which matches well with the standard data of JCPDS card no. 79-0207. No additional diffraction peaks of other impurities (such as Co, CoO, or Co_3_O_4_ phases) were detected. These results suggest that Co^2+^ substitute the sites of Zn^2+^ in the ZnO lattice without changing the crystalline structure of ZnO host. Fig. 1(b) shows the three primary peaks of (100), (002) and (101) planes for all the samples. The peak intensity is decreased and peak width is broaden by doping Co^2+^, which is probably attributed to the lattice disorder and internal stress induced by doping Co^2+^.^36^ The average grain sizes and lattice parameters are summarized in Table 1. Compared with the undoped ZnO, the slight red shift of diffraction peaks and reduced lattice constants are mainly due to that the ionic radius of Co^2+^ (0.72 Å) is smaller than that of Zn^2+^ (0.74 Å). ICP-AES was used to characterize the chemical compositions of all the samples, which are also summarized in Table 1. The content of Co dopant in the Co-doped ZnO nanoparticles increases as the amount of original Co precursor increases. The Co content in the samples is about two time larger that their molar ratios in the precursors. This is probably due to that Co(acac)_2_ is easier decomposed than Zn(acac)_2_.

**Table 1:** Summaries of Co/Zn ratio, particles size (*D*), grain size (*G*) and lattice constants of Zn_1−*x*_Co_*x*_O nanoparticles (0 ≤ *x* ≤ 0.04)

Zn_1−*x*_Co_*x*_O	Co/Zn^a^	*D*/nm	*G*/nm	Lattice constants		
				*a*/Å	*c*/Å	*c*/*a*
*x* = 0	0%	15.2	15.6	3.2580	5.2317	1.6058
*x* = 0.01	2.2%	12.9	13.3	3.2595	5.2128	1.5993
*x* = 0.02	3.3%	14.8	14.5	3.2592	5.2039	1.5967
*x* = 0.04	8.9%	11.9	12.7	3.2560	5.2132	1.6011

a The Co/Zn ratio is examined by ICP-AES measurement.

**Fig. 2:**
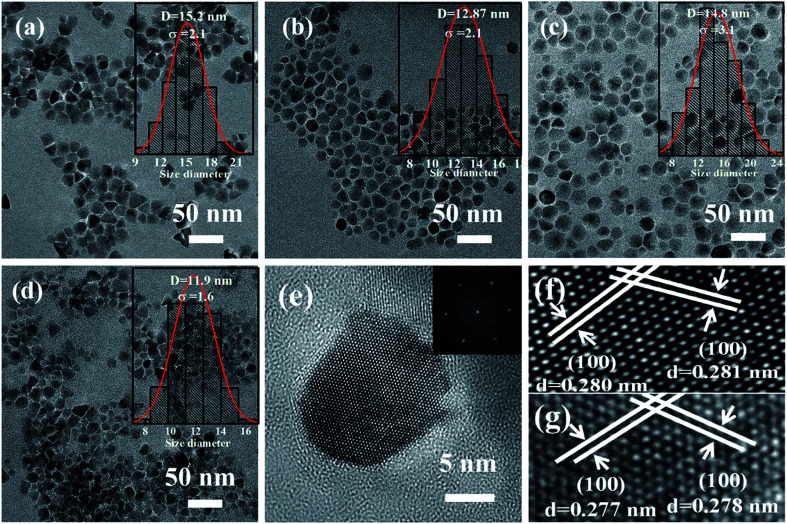
TEM images of Zn_1−*x*_Co_*x*_O nanoparticles: (a) *x* = 0, (b) *x* = 0.01, (c) *x* = 0.02 and (d) *x* = 0.04. Insets show the related size distributions; (e) TEM image of a single Zn_0.98_Co_0.02_O nanoparticle. Inset shows the corresponding FFT pattern; HRTEM images of (f) undoped ZnO and (g) Zn_0.98_Co_0.02_O nanoparticles.

Fig. 2(a–d) displays the TEM images of Zn_1−*x*_Co_*x*_O nanoparticles with different Co doping concentrations. All the samples show nearly spherical shape with some triangles, and non-agglomeration phenomenon happens among the particles. To obtain the average particle size, we did a survey of at least 200 nanoparticles for each sample. The statistical results show that all the samples have a narrow size distribution. The particle sizes of Zn_1−*x*_Co_*x*_O nanoparticles are in the range of 11–15 nm, as summarized in Table 1. These results indicate that the Co dopants have little impact on the size of products. These Co-doped ZnO nanoparticles with negligible size difference are promised to be good candidates to explore the intrinsic nature of ferromagnetic ordering in DMSs, for we can avoid the influence of particle size and aggregation on the magnetic properties of nanostructures. The average particle size of each sample is in good agreement with the grain size calculated based on XRD patterns, which indicates that each individual nanoparticle is a single nanocrystal. Fig. 2(f) and (g) show the representative HRTEM images of undoped ZnO and Zn_0.98_Co_0.02_O nanoparticles, respectively, which show clearly atomic lattice fringes. For both the samples, the interplanar spacing distance is about 0.28 nm with a separation angle of 120°, corresponding to the (100) plane of ZnO. Moreover, compared with the undoped ZnO nanoparticles (Fig. 2(f)), the interplanar spacing distance of Co-doped ZnO nanoparticles (Fig. 2(g)) is slightly decreased. This is mainly resulted from that part of Zn^2+^ sites in the ZnO lattice were replaced by Co^2+^ with smaller ionic radius, which is accordant with the XRD results.

**Fig. 3:**
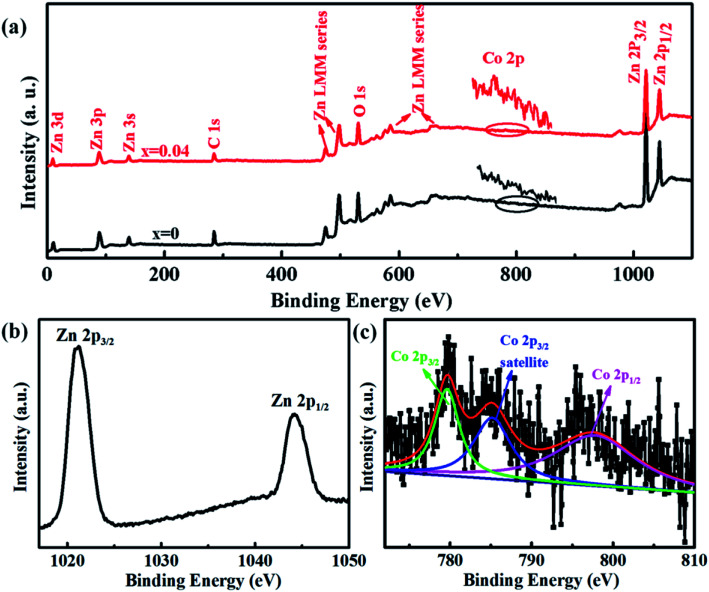
XPS spectra of undoped ZnO and Co-doped ZnO nanoparticles: (a) full scan spectra; (b) Zn 2p spectrum; (c) Co 2p spectrum.

The electronic and chemical states of undoped ZnO and Co-doped ZnO nanoparticles were investigated by XPS measurements. In comparison with the undoped ZnO nanoparticles, the additional Co signal confirms the existence of Co element in the Co-doped ZnO nanoparticles (Fig. 3(a)). The high-resolution of Zn 2p spectrum (Fig. 3(b)) shows two peaks located at 1021.1 and 1044.2 eV, corresponding to Zn 2p_3/2_ and Zn 2p_1/2_, respectively. These two peaks reveal a spin-orbital splitting of 23.1 eV, indicating the existence of Zn^2+^ oxidation state in the samples.^4,37^ The high-resolution Co 2p spectrum of Co-doped ZnO nanoparticles (Fig. 3(c)) can be fitted by three peaks. The two peaks located at 779.7 and 795.6 eV with a split spin–orbit component of 15.9 eV can be ascribed to the Co 2p_3/2_ and Co 2p_1/2_, respectively.^23^ The other peak at 785.2 eV can be attributed to the satellite peak of Co 2p_3/2_. Obviously, the binding energy of satellite peak is about 5.5 eV larger than that of the main peak of Co 2p_3/2_, suggesting that the Co dopants exist in the nanoparticles as Co^2+^.^38^

**Fig. 4:**
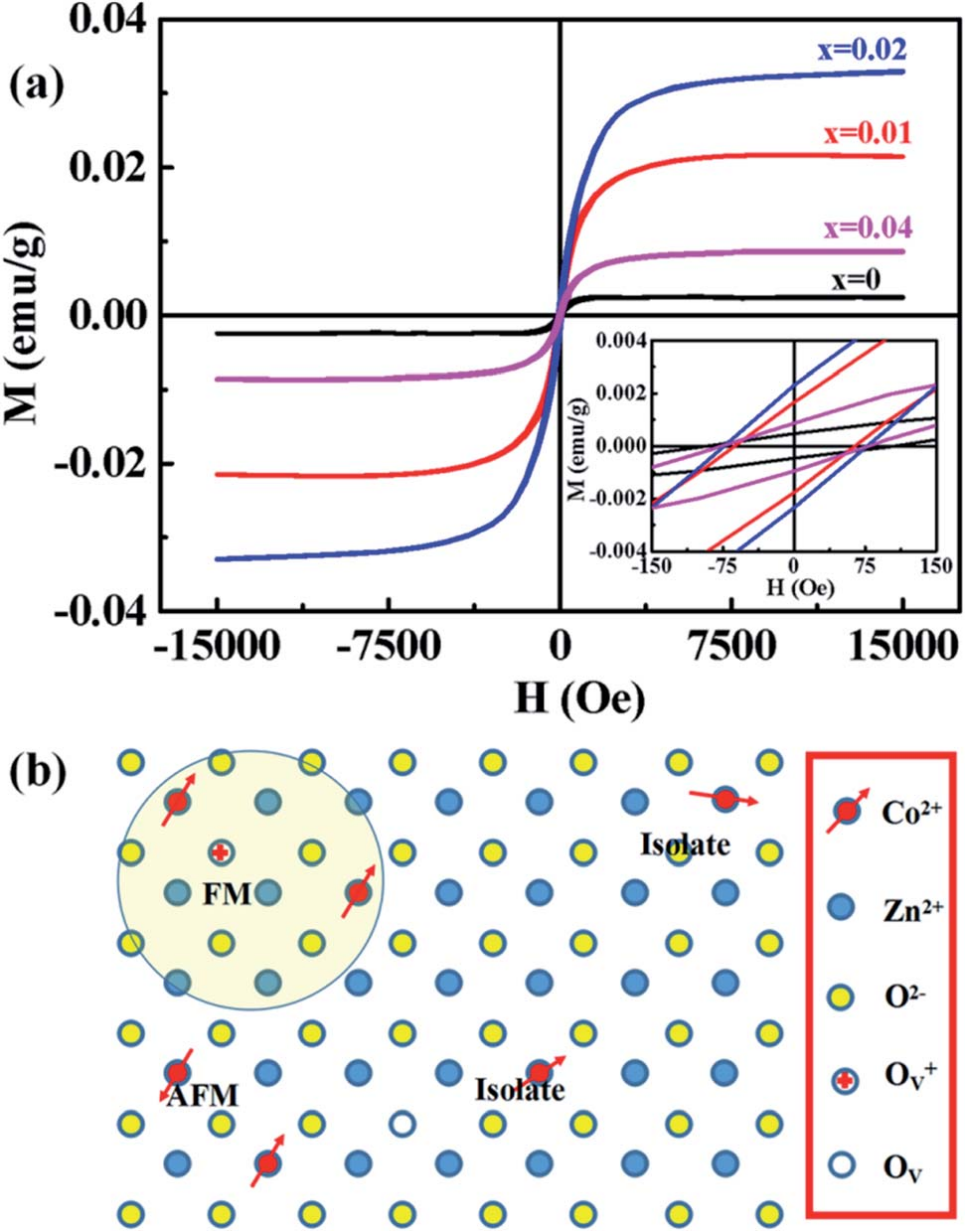
(a) M–H curves of Zn_1−*x*_Co_*x*_O nanoparticles (0 ≤ *x* ≤ 0.04) at room temperature. Inset is the amplification of centre part. (b) Schematic illustration of BMP formation in Co-doped ZnO nanoparticles driven by oxygen vacancies and Co^2+^ dopants.

Magnetic hysteresis (M–H) loops of undoped and Co-doped ZnO nanoparticles are measured at room temperature, as shown in Fig. 4(a). After the paramagnetic and antiferromagnetic signals were deducted, all the samples present S-like curves, showing room temperature ferromagnetic (RTFM) characteristic. The magnetic parameters, including saturation magnetization (*M*_s_), remanence (*M*_R_) and coercivity (*H*_C_) are summarized in Table 2. The ferromagnetic behavior of undoped ZnO nanoparticles with a *M*_s_ value of 0.0025 emu g^−1^ is originated from the oxygen defects with unpaired electron spins, which has been demonstrated in our previous work.^4^ When Co ions are incorporated within the ZnO crystal lattice, the *M*_s_ values abruptly increase to 0.0329 emu g^−1^ with 2% Co concentration, and then decrease to 0.0087 emu g^−1^ with 4% Co concentration. The variation of Ms *versus* Co^2+^ doping concentration should be caused by the formation of long-range ferromagnetic ordering driven by defect-mediated bound magnetic polarons (BMPs).^33,34,39^ We assume that the Co^2+^ dopant may induce oxygen vacancies with single positive charge 
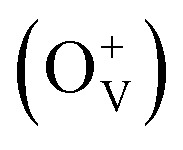
, which can serve as localized defects in the lattice of Co-doped ZnO nanoparticles. Fig. 4(b) shows the BMP formation in Co-doped ZnO nanoparticles driven by oxygen vacancies and Co^2+^ dopants. The electron trapped in oxygen vacancies can induce spin orientation for the adjacent Co^2+^. With a relative lower Co^2+^ concentration, the spin orientations of the two neighboring Co^2+^ are on the same direction, forming ferromagnetic coupling of 
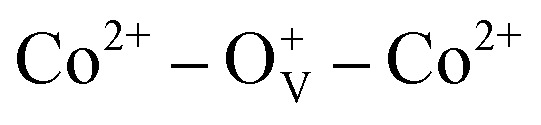
. When a higher Co^2+^ concentration is doped in the ZnO host, the extra Co^2+^ locates in the O^2−^ lattice, forming antiferromagnetic coupling of Co^2+^–O^2−^–Co^2+^, which can greatly reduce the magnetic moments.

**Table 2:** The relationship between doped Co concentration, oxygen defects and magnetic parameters for undoped and Co-doped ZnO nanoparticles

Samples	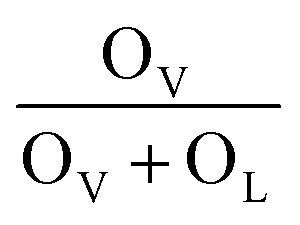	*M*_s_ (emu g^−1^)	*M*_R_ (emu g^−1^)	*H*_c_ (Oe)
ZnO	30.1%	0.0025	0.00047	99.3
Zn_0.99_Co_0.01_O	44.2%	0.0215	0.00172	63.1
Zn_0.98_Co_0.02_O	53.6%	0.0329	0.00233	75.6
Zn_0.96_Co_0.04_O	29.7%	0.0087	0.00091	77.3
Zn_0.98_Co_0.02_O–A^a^	11.3%	0.0252	0.0034	99.6

a The Zn_0.98_Co_0.02_O nanoparticles annealed in air at 200 °C for 2 h is marked as Zn_0.98_Co_0.02_O–A.

**Fig. 5:**
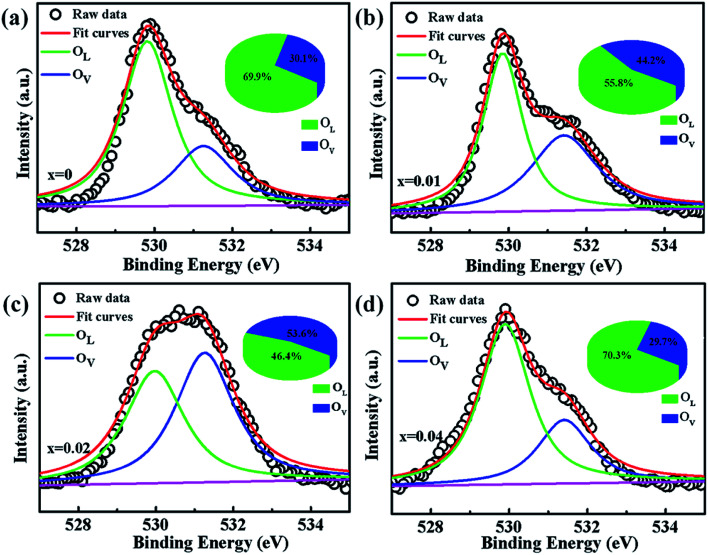
High resolution O 1s XPS spectra of Zn_1−*x*_Co_*x*_O nanoparticles: (a) *x* = 0, (b) *x* = 0.01 (c) *x* = 0.02 and (d) *x* = 0.04.

To verify if the intrinsic nature of ferromagnetic coupling in Co-doped ZnO nanoparticles is induced by oxygen defects as assumed above, high resolution XPS measurements were adopted to characterize the situation of oxygen, as displayed in Fig. 5. The high resolution O 1s XPS spectra of all the samples can be fitted by two peaks, the one located at 529.9 eV (marked by O_L_) is attributed to the lattice O^2−^ anion in the Zn–O bond and the other one at 531.4 eV (marked by O_V_) is related to the oxygen defects.^4,40^ The relative concentration of oxygen vacancies (O_V_/(O_V_ + O_L_)) can be roughly semiquantitative by its area ratio in the O 1s XPS curves, which is expressed as sector diagrams in the inset of spectra. The concentration of oxygen vacancies in the Co-doped ZnO nanoparticles increases first and then decreases by improving the doping Co^2+^ concentration, which is consistent with the tendency of *M*_s_ (Fig. 4). There results suggest that the doping Co^2+^ can result in more oxygen vacancies, which is beneficial for the formation of ferromagnetic coupling and improvement of magnetism in ZnO.

**Fig. 6:**
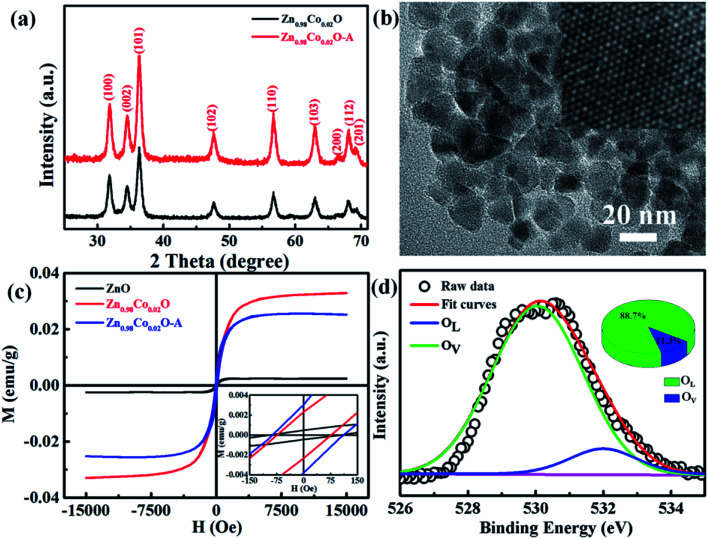
(a) XRD patterns and (b) TEM image of the Zn_0.98_Co_0.02_O nanoparticles annealed at 200 °C in air. Inset of (b) shows the HRTEM image. (c) M–H curves for undoped ZnO, original Zn_0.98_Co_0.02_O and Zn_0.98_Co_0.02_O–A. Inset shows the amplification of centre part for M–H curves. (d) High resolution O 1s XPS spectrum of Zn_0.98_Co_0.02_O nanoparticles annealed at 200 °C in air.

In order to further investigate the effect of doping Co^2+^ and oxygen vacancies on the magnetic properties of Co-doped ZnO nanoparticles, we annealed the Zn_0.98_Co_0.02_O nanoparticles with the highest *M*_s_ value in air at 200 °C for 2 h (named as Zn_0.98_Co_0.02_O–A), and analyzed its magnetic properties. Fig. 6(a and b) shows the XRD pattern and TEM image of the Zn_0.98_Co_0.02_O annealed at 200 °C in air. Compared with the original Zn_0.98_Co_0.02_O nanoparticles (Fig. 1 and 2), the air-annealing treatment doesn't change its crystalline structure and morphology, which can eliminate their possible affects on magnetic properties. M–H curves (Fig. 6(c)) clearly show that the *M*_s_ value decreases from 0.0329 to 0.0252 emu g^−1^ after air-annealing treatment. Comparison of the high resolution O 1s XPS spectra (Fig. 5(c) and 6(d)) reveals that the concentration of oxygen vacancies decreases from 53.6% to 11.3% after air-annealing treatment. This phenomenon further demonstrates that the oxygen vacancies can greatly improve the magnetic properties of Co-doped ZnO nanoparticles. Besides, the *M*_s_ value of Zn_0.98_Co_0.02_O after air-annealing treatment is still larger than the undoped ZnO nanoparticles (Fig. 6(a)), although its concentration of oxygen vacancies is only 11.3% which is lower than the undoped one. This result suggests that the Co^2+^ dopant also plays an important role on the magnetic improvement of ZnO.

To further analyze the effects of doping Co atoms and oxygen defects on the magnetization, all the M–H curves were fitted by BMP's model,^10,41–43^ as shown in Fig. S1 and S2.† The relative fitted parameters are listed in Table S1.† The effective spontaneous magnetic moment per BMP (ms) decrease and then increase with the enhancement of Co atoms, which are accordant with the tendency of oxygen defects. The spontaneous magnetic moment per BMP (ms) firstly decrease and then increase by increasing the concentrations of Co dopant. This indicates that doping Co can create more oxygen defects and decrease the value of ms at a relative low concentration of Co dopant. By further increasing the concentration of Co dopant, the dopant concentration exceeds the related percolation threshold, leading to the increase of number of BMP and decrease of the effective spontaneous magnetic moment per BMP. These results indicate the coefficient effect of doping effect and oxygen effect on the magnetization.

**Fig. 7:**
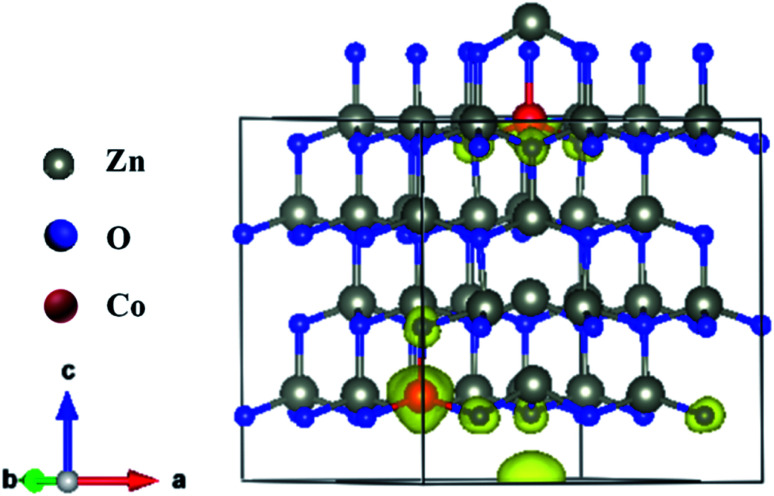
Spin density distribution in 3 × 3 × 2 ZnO supercell doped by two Co^2+^ with two single charge oxygen vacancies.

The magnetism in Co-doped ZnO system is calculated using density functional theory (DFT). The detailed calculated process is described in the ESI.† According to the experimental model, a 3 × 3 × 2 ZnO supercell with two Zn atoms replaced by Co atoms was constructed, which has two oxygen vacancies. Fig. S3† shows the original structure of 3 × 3 × 2 ZnO supercell with two doped Co atoms and two oxygen defects without optimization. Before the magnetism calculation, we established the supercells with different type of oxygen defects, including single charged oxygen vacancies 
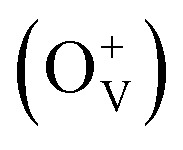
 and neutral oxygen vacancies. Optimization of these supercells reveals that the system with 
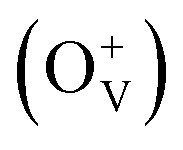
 has the lowest formation energy and performs ferromagnetism, while the O_v_ system shows higher formation energy and antiferromagnetism (Table S2†). Fig. 7 shows the spin-density distribution of Co-doped ZnO with two single charge oxygen vacancies. The spin-density focuses on the Co atoms sites, which originates from the unpaired d electron of Co ions. The spin density of Co ions is positive in the Co-doped ZnO, indicating the presence of ferromagnetic coupling between Co atoms *via* single charge oxygen defects.

**Fig. 8:**
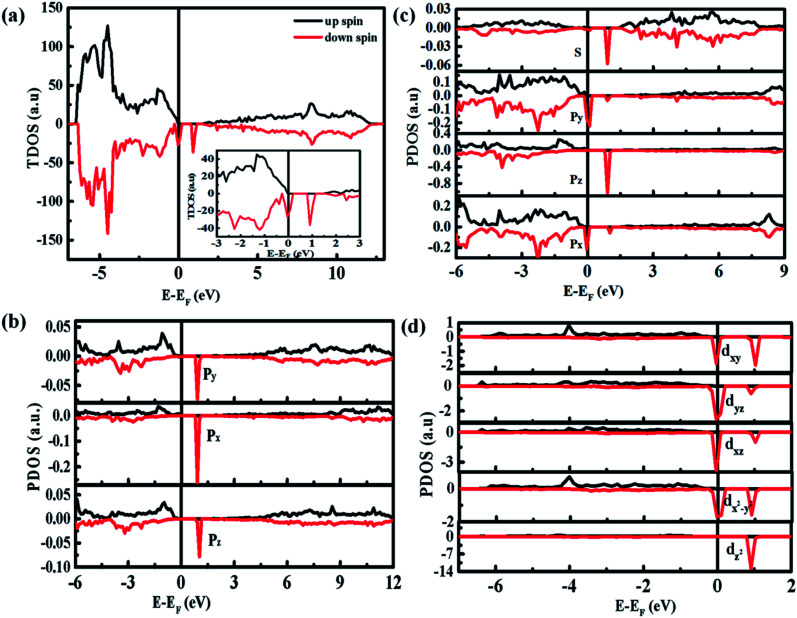
Calculated DOS spectra of the 3 × 3 × 2 ZnO supercell doped by two Co^2+^ with two single charge oxygen vacancies: (a) total DOS. Inset shows the corresponding magnification near *E*_F_; (b) partial DOS of O 2s and O 2p surrounding with Co ions; (c) 3p and (d) 3d of Co ions surrounding with two single charge oxygen vacancies.

Fig. 8 shows the densities of states (DOS) spectra of the 3 × 3 × 2 ZnO supercell doped by two Co^2+^ with two single charge oxygen vacancies. The total DOS spectrum (Fig. 8(a)) reveals that the up and down spin states near the Fermi energy (*E*_F_) level are asymmetry, suggesting the existence of spin polarized state, which may induce ferromagnetic ordering.^44^ Compared with the DOS spectra of undoped ZnO,^4^ the spin polarized state is closed to the bottom of valence band, which is due to the doping Co^2+^. The partial DOS spectrum of 3d orbits for Co ions (Fig. 8(d)) and 2p orbits for O atom surrounding Co ions (Fig. 8(b)) are also asymmetry near the Fermi energy (*E*_F_) level, indicating that the natural ferromagnetism mainly originates from the 3d orbits of Co ions with a local spin moment of ∼2.71 *μ*_B_ and 2p orbits of O ions surrounding Co ions with a weaker local spin moment of ∼0.15 *μ*_B_. Compared with the 2p orbits of O atoms and 3d orbits of Co atoms, the 2s orbits of O atoms and 3p orbits of Co atoms can only provide 0.006 and 0.054 *μ*_B_, respectively, which can be ignored. Furthermore, due to the existence of spin polarization at the same energy, the p–d interaction exists between Co atoms and their surrounding O atoms. The ZnO supercell doped by four Co^2+^ with two single charge oxygen defects also reveal the same results (Fig. S4 and S5†).

## Conclusion

In summary, we reported a thermal decomposition method for the fabrication of Co-doped ZnO nanoparticles with tunable Co^2+^ doping concentrations, which show room-temperature ferromagnetic characteristic. The Co^2+^ doping effect on the structure, morphology and magnetic performance of ZnO nanoparticles were investigated. The doping Co^2+^ has little influence on the structure and morphology of ZnO nanoparticles, but can effectively increase the oxygen defects and boost the magnetic properties. Air annealing experiment further verify the great significance of oxygen defects on the magnetism. Density functional theory calculation suggests that the long-range magnetic ordering in Co-doped ZnO nanoparticles is originated from the localized spin moments of 3d electrons of Co^2+^ ions. Experimental and theoretical studies indicate that the ferromagnetism in Co-doped ZnO system is attributed to the formation of oxygen defect-induced bound magnetic polarons, during which 
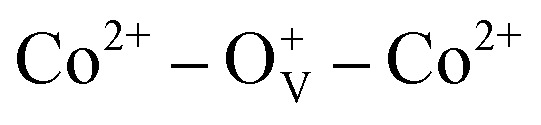
 ferromagnetic coupling can be introduced by the doping Co^2+^ and oxygen defects. These findings give an insight into the intrinsic magnetic nature of diluted magnetic semiconductors from an experimental and theoretical view, and provide a guidance to control their magnetic properties, which is significant for the spintronics and spin-electron devices.

## Conflicts of interest

There are no conflicts to declare.

## Supplementary Material

RA-009-C9RA03620B-s001
