# Supplementary material for: Doping effect and oxygen defects boost room temperature ferromagnetism of Co-doped ZnO nanoparticles: experimental and theoretical studies
Source: RSC Adv. 2019 Jul 25;9(40):23012–20. doi: 10.1039/c9ra03620b (PMC9067132; doi:10.1039/c9ra03620b)
Supplement: RA-009-C9RA03620B-s001 [file RA-009-C9RA03620B-s001.pdf]

Electronic Supplementary Material (ESI) for RSC Advances.
This journal is © The Royal Society of Chemistry 2019

Supporting Information

Doping effect and oxygen defect boost room temperature ferromagnetism of Co-doped ZnO nanoparticles: experimental and theoretical studies

Yan Zong[a1], Yong Sun[a1], Shiyan Meng[a], Yajing Wang[a], Hongna Xing[a], Xinghua Li[a]*, Xinliang Zheng[a]

[a] School of Physics, Northwest University, Xi'an, 710127, China

Yan Zong and Yong Sun contribute equally to this work

*Corresponding authors: X. Li (xinghua.li@nwu.edu.cn)

Calculation details

The magnetism of Co doped ZnO systems with oxygen vacancies is estimated by the fundament of the density functional theory (DFT).[1] The generalized gradient approximation (GGA) with spin-polarized Perdew-Burke-Ernerhof (PBE) method was performed for the calculation of exchange and correlation functional.[2] The core electrons were represented by the projector augmented-wave (PAW) potential.[3]

In comparison with the experimental results, the basic ZnO system with a 3×3×2 supercell containing 70 atoms (36 Zn atoms and 34 O atoms) was used to calculate the related parameter of magnetic properties. Based on this system, some Zn atoms in supercell were replaced by Co atoms, and some oxygen atoms were removed according to the experimental model. In addition, the Brillouin zone integrations were performed using a 3×3×2 Monkhorst-Pack *k*-grid and cutoff energy was 400 eV.[4] Self-consistency was achieved when the total energy converged to 1.0×10^{-4} eV per atom and the Hellmann-Feynman force on an atom was smaller than 0.01 $eVÅ^{-1}$. The calculated lattice constants for bulk ZnO were a = b = 3.287 Å and c = 5.301 Å, which are in good accordance with the experiment values (a = b = 3.25 Å and c = 5.22 Å).

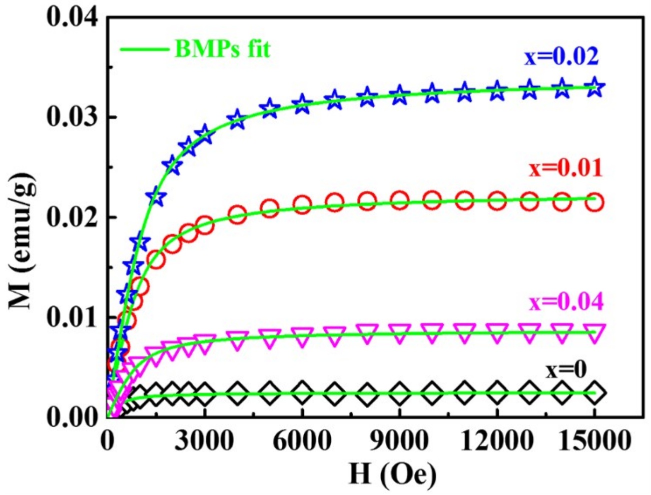

Fig. S1 M-H curves fitted with BMP model for undoped and Co-doped ZnO samples.

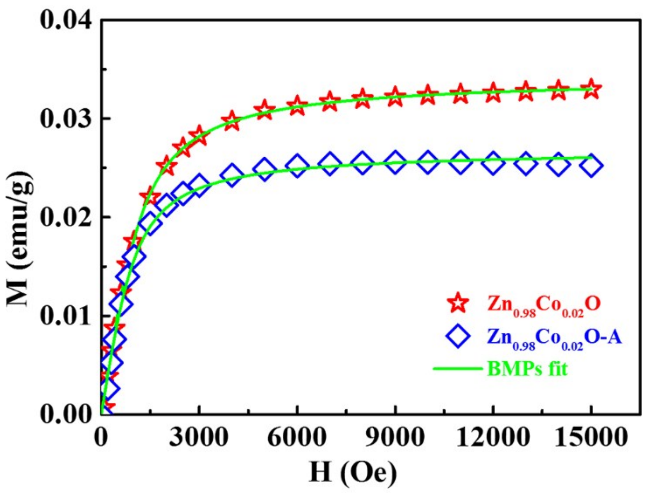

Fig. S2 M-H curves fitted by BMP model for original $Zn_{0.98}Co_{0.02}O$ and $Zn_{0.98}Co_{0.02}O$-A.

To Further analyze the effects of doping Co atoms and oxygen defects on the magnetization, the M-H curves were fitted according to the Langevin function[5-8]:

$$M = nm_s\left[\coth\frac{m_{eff}H}{K_BT} - \frac{K_BT}{m_{eff}H}\right] + \chi_m H \tag{1}$$

where n, m_s and m_{eff} are number of BMP, 'effective' spontaneous magnetic moment per BMP and true spontaneous magnetic moment per BMP. The first term in equation

($nm_s\left[\coth\frac{m_{eff}H}{K_BT}-\frac{K_BT}{m_{eff}H}\right]$) represents the contribution of BMP's model. The second term ($\chi_m H$) is the matrix contribution, which is equal to the paramagnetic contribution from original magnetic data. This part has no influence on the BMP's model. Herein, the paramagnetic signals have been subtracted. Therefore, the M-H curves were fitted by the following equation according to the BMP model:

$$M = nm_s\left[\coth\frac{m_{eff}H}{K_BT}-\frac{K_BT}{m_{eff}H}\right] \quad (2)$$

Fig. S1 shows the M-H curves fitted with BMP model for undoped and Co-doped ZnO samples. Fig. S2 shows the M-H curves fitted by BMP model for original $Zn_{0.98}Co_{0.02}O$ and $Zn_{0.98}Co_{0.02}O$-A. Obviously, the M-H curves can be well fitted by BMP model. The related fitted parameters are listed in Table S1.

Table S1 Parameters obtained from BMP fitting for undoped and Co-doped ZnO nanoparticles

Samples	Ms (emu/g)	ms (*10^3 μ_B)	N(*10^{16}/cm^3)
ZnO	0.0025	22.02	1.219
$Zn_{0.99}Co_{0.01}O$	0.0215	10.55	23.06
$Zn_{0.98}Co_{0.02}O$	0.0329	8.51	43.42
$Zn_{0.96}Co_{0.04}O$	0.0087	11.16	8.485
$Zn_{0.98}Co_{0.02}O$-A[a]	0.0252	10.27	28.23

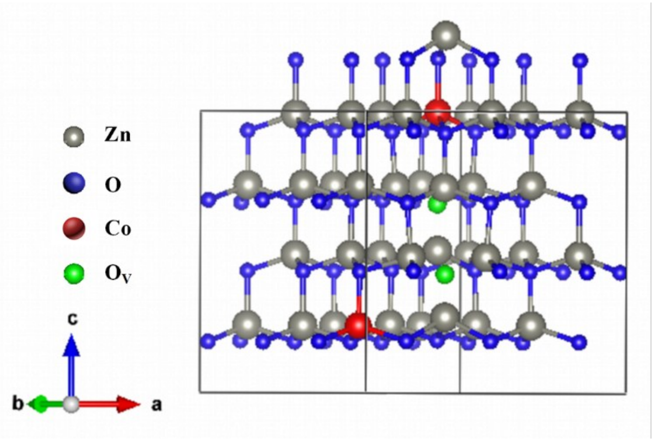

Fig. S3 The original structure of 3×3×2 ZnO supercell with two doped Co atoms and two oxygen defects without optimization.

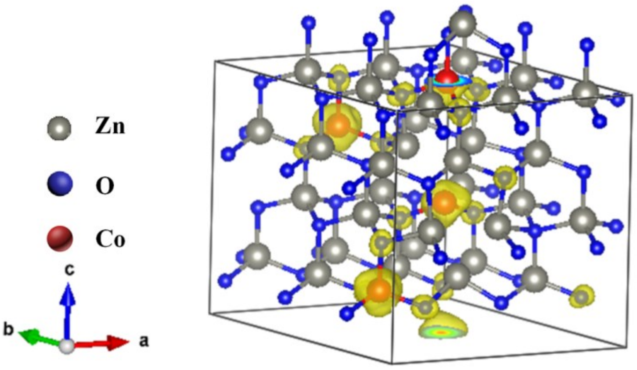

Fig. S4 Spin density distribution with two single charge oxygen vacancies in 3×3×2 ZnO supercell doped by four Co^{2+}.

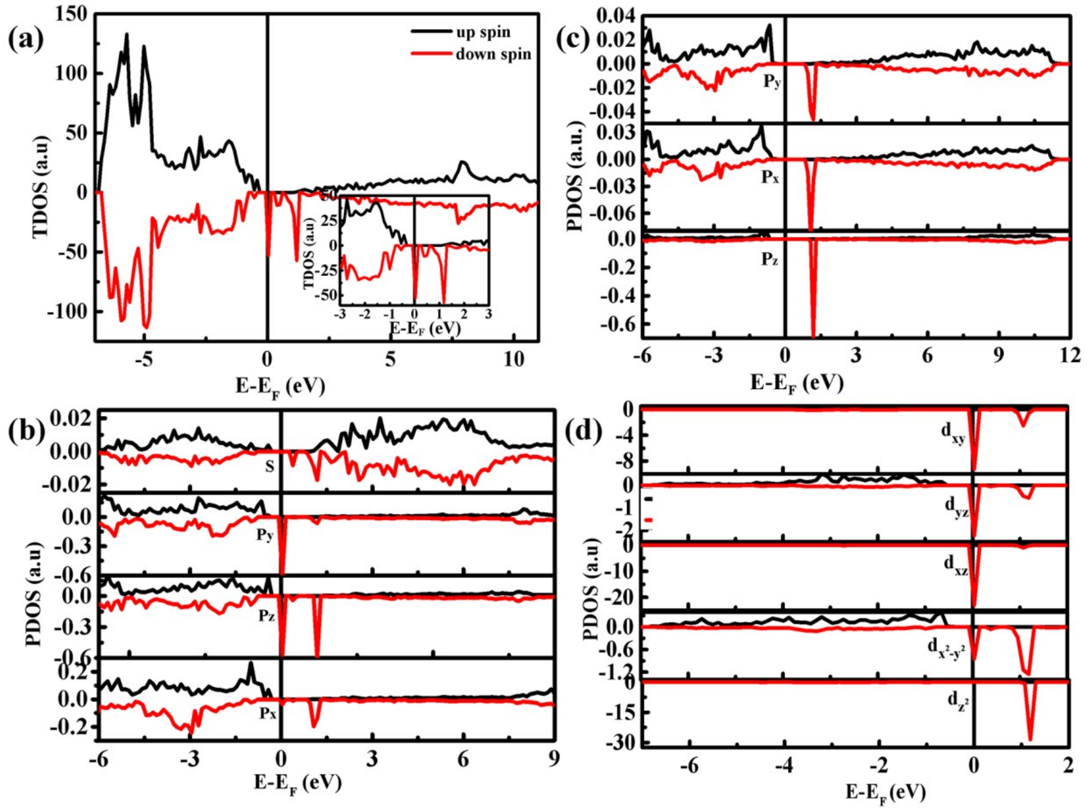

Fig. S5 Calculated DOS spectra of the 3×3×2 ZnO supercell doped by four Co^{2+} with two single charge oxygen vacancies: (a) total DOS. Inset shows the corresponding magnification near E_F.; (b) partial DOS of O 2s and O 2p surrounding with Co ions; (c) 3p and (d) 3d of Co ions surrounding with two single charge oxygen vacancies.

Table S2 Comparison of formation energy and magnetic moment for the Co-doped 3×3×2 ZnO lattice (2 Co atoms and 34 Zn atoms) with different oxygen defect systems

Oxygen defect states	Magnetic state	Formation energy	Magnetic moment (μ_B)
	AFM	-0.32241700E+03	-0.0246
$2Ov^+$	**FM**	**-0.32242331E+03**	**8.004**
	PM	-0.31987527E+03	--
	AFM	-0.32101104E+03	-0.0318
Ov^++Ov	FM	-0.32096158E+03	6.9988
	PM	-0.31831664E+03	--
	AFM	-0.31879413E+03	0.0062
2Ov	FM	-0.31879371E+03	6.0033
	PM	-0.31601803E+03	--

References

1. G. Kresse and J. Furthmuller, *Phys. Rev. B*, 1996, **54**, 11169-11186.

2. J. P. Perdew and Y. Wang, *Phys. Rev. B*, 1986, **33**, 8822-8824.

3. G. Kresse and J. Joubert, *Phys. Rev. B*, 1999, **59**, 1758-1775.

4. H. J. Monkhorst and J. D. Pack, *Phys. Rev. B*, 1976, **13**, 5188-5192.

5. A. Nasir, S. Budhi, A. K. Zaheer, A. R. Vijaya, T. Kartick and G. Subhasis, *Sci. Rep-UK*, 2019, **9**, 2461.

6. G. H. McCabe, T. Fries, M. T. Liu, and Y. Shapira, *Phys. Rev. B*, 1997, **56**, 6673-6680.

7. N. Karak, B. Pal, D. Sarkar and T. K. Kundu, *J. Alloy. Compd.*, 2015, **647**, 252-258.

8. V. R. Akskay, B. Arun, G. Mandal, G. R. Mutta and A. Chanda, J. Phys. Chem. C,

2018, **122**, 26592-26604.

9. J. M. D. Coey, M. Venkatesan and C. B. Fitzgerald, *Nat. Mater.*, 2005, **4**, 173-179.
